# Supplementary material for: Histone Acetylation Dynamics during In Vivo and In Vitro Oocyte Aging in Common Carp Cyprinus carpio
Source: Int J Mol Sci. 2021 Jun 3;22(11):6036. doi: 10.3390/ijms22116036 (PMC8199789; doi:10.3390/ijms22116036)
Supplement: Supplementary file 1 [file ijms-22-06036-s001.zip › Supplementary file 2.pdf]

Carp histone new

Experiment: Carp histone new

Report created: 11-11-2020 10:40:53

Reference image

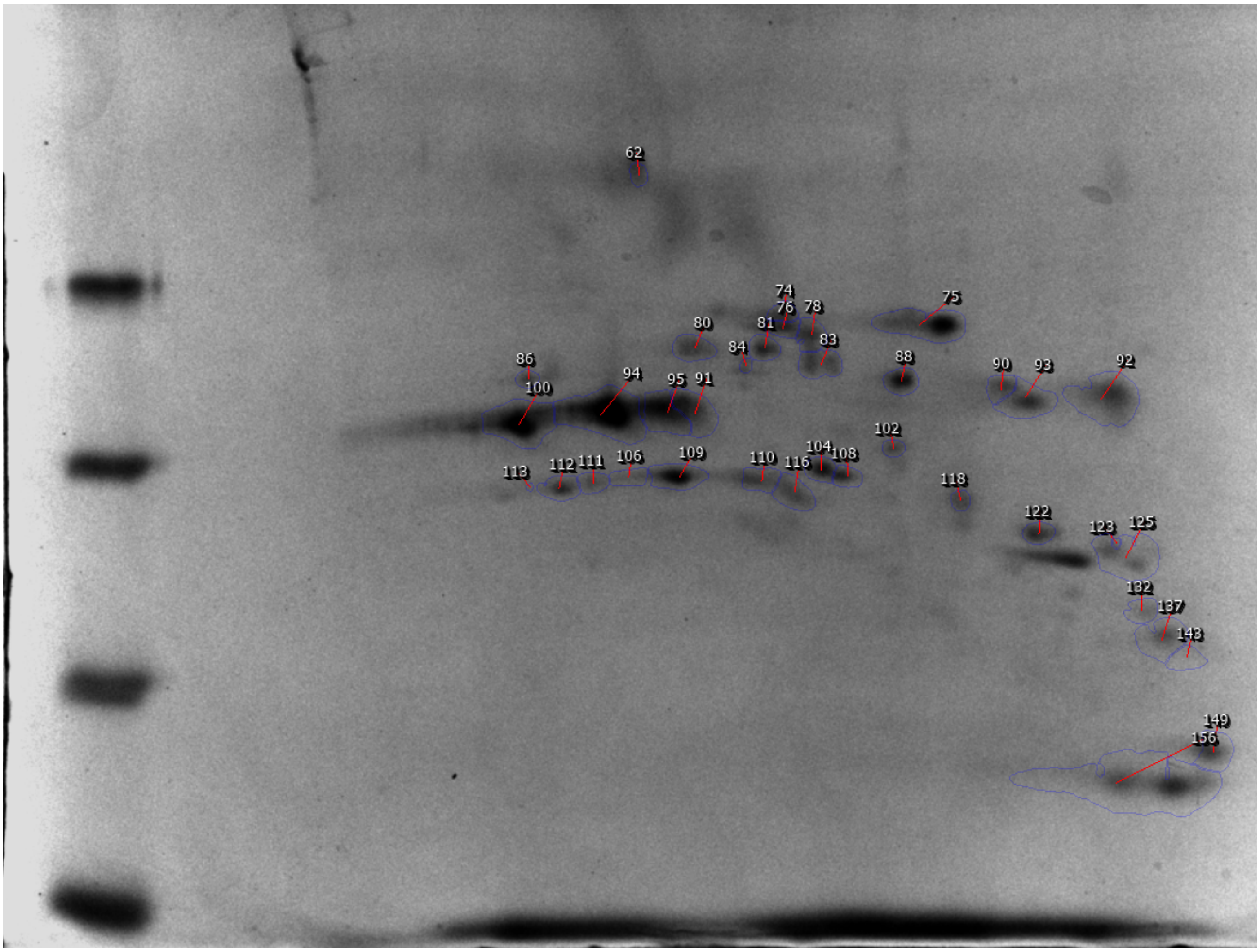

Experiment Design

Group	Control O HPS	In vitro 8 HPS
Replicates	5	5

Spots

#	Anova (p)	Fold	Notes	Average Normalised Volumes	
				Control O HPS	In vitro 8 HPS
62	0,136	1,3		8998,352	7080,193
74	0,766	1,1		1,714e+004	1,856e+004
75	0,525	1,4		2,359e+005	1,728e+005
76	0,194	1,5		1,723e+004	2,657e+004
78	0,187	1,3		2,998e+004	3,840e+004
80	0,206	1,1		3,423e+004	3,005e+004
81	0,284	1,3		2,031e+004	2,642e+004
83	0,251	1,3		3,552e+004	4,596e+004
84	0,558	1,1		3245,137	3664,910
86	0,933	1,1		1,073e+004	1,214e+004
88	0,342	1,3		3,158e+004	4,012e+004
90	0,345	1,7		4,107e+004	2,488e+004
91	0,337	1,1		9,757e+004	8,635e+004
92	0,490	1,2		2,196e+005	1,802e+005
93	0,773	1,3		1,318e+005	1,022e+005
94	0,558	1,2		4,654e+005	3,954e+005
95	0,510	1,2		1,387e+005	1,143e+005

#	Anova (p)	Fold	Notes	Average Normalised Volumes	
				Control O HPS	In vitro 8 HPS
100	0,560	1,2		2,909e+005	2,355e+005
102	0,760	1,1		8859,709	9566,886
104	0,613	1,1		3,241e+004	3,615e+004
106	0,372	1,1		1,699e+004	1,937e+004
108	0,477	1,1		2,134e+004	2,389e+004
109	0,994	1,0		1,022e+005	1,048e+005
110	0,265	1,2		4,078e+004	3,431e+004
111	0,706	1,1		1,938e+004	1,823e+004
112	0,928	1,0		4,437e+004	4,341e+004
113	0,086	1,6		648,064	1021,671
116	0,621	1,1		4,571e+004	4,297e+004
118	0,607	1,1		7419,333	7918,445
122	0,711	1,2		2,126e+004	2,446e+004
123	0,823	1,7		1246,105	2066,391
125	0,910	1,1		1,413e+005	1,290e+005
132	0,945	1,1		2,563e+004	2,427e+004
137	0,801	1,1		8,519e+004	7,580e+004
143	0,444	1,3		3,060e+004	2,385e+004
149	0,208	1,5		1,027e+005	7,074e+004
156	0,505	1,3		8,279e+005	6,233e+005

Identifier 62

Position (977, 261)
Notes

Control O HPS | In vitro 8 HPS

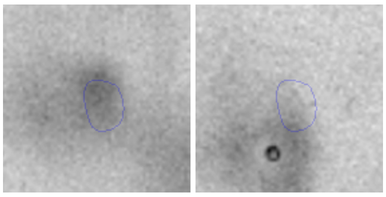

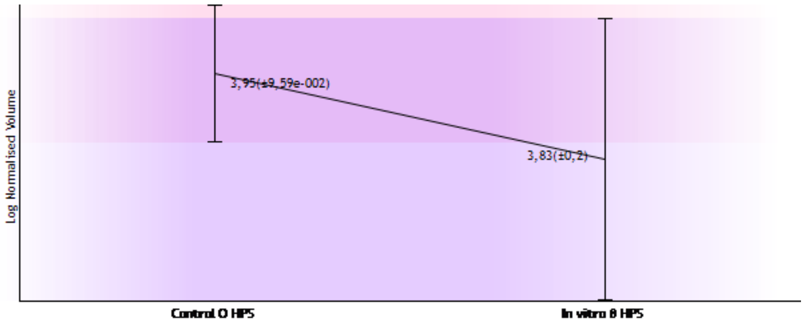

Identifier 74

Position (1199, 483)
Notes

Control O HPS | In vitro 8 HPS

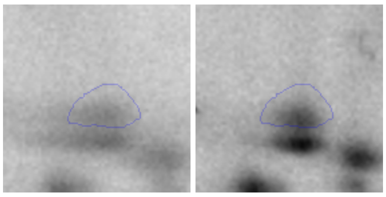

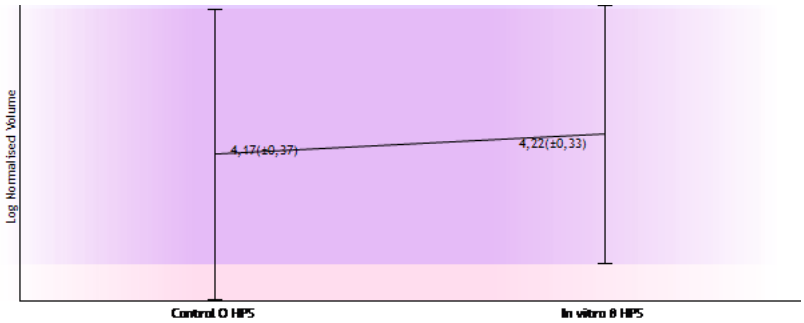

Identifier 75

Position (1443, 492)
Notes

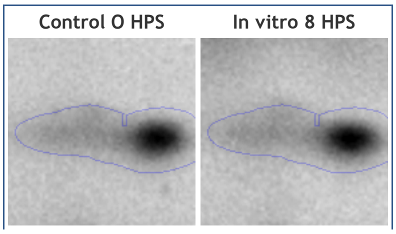

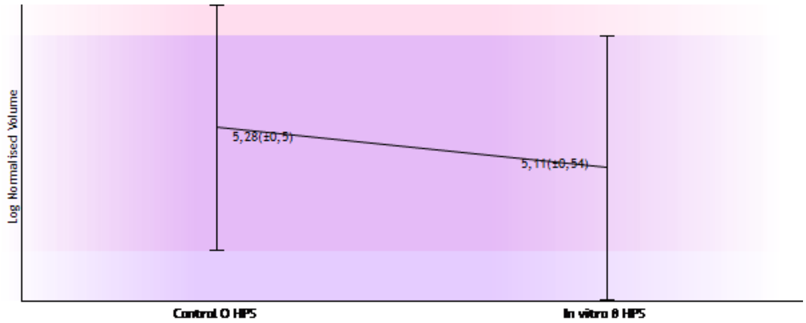

Identifier 76

Position (1199, 495)
Notes

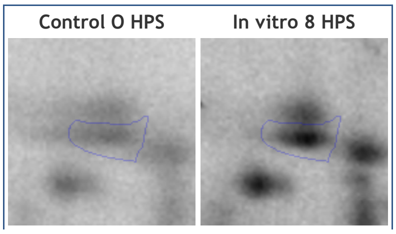

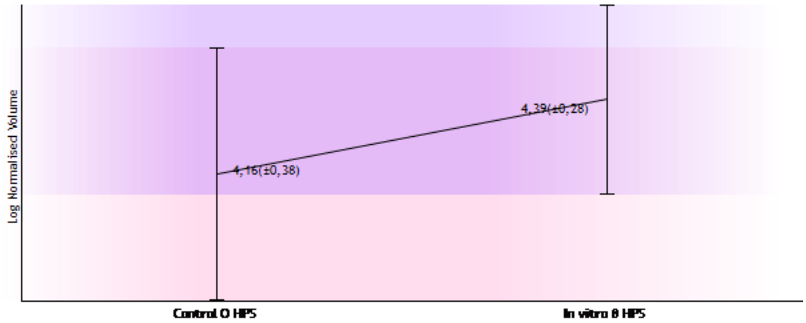

Identifier 78

Position (1239, 507)
Notes

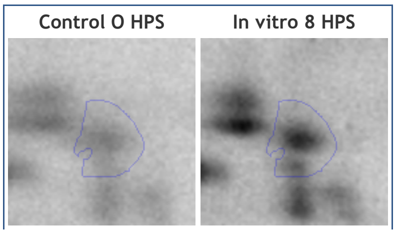

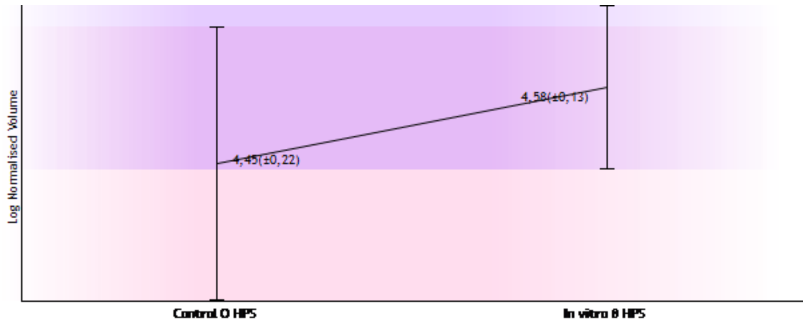

Identifier 80

Position (1051, 528)
Notes

Control O HPS	In vitro 8 HPS

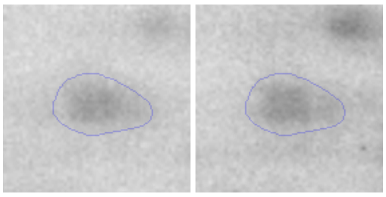

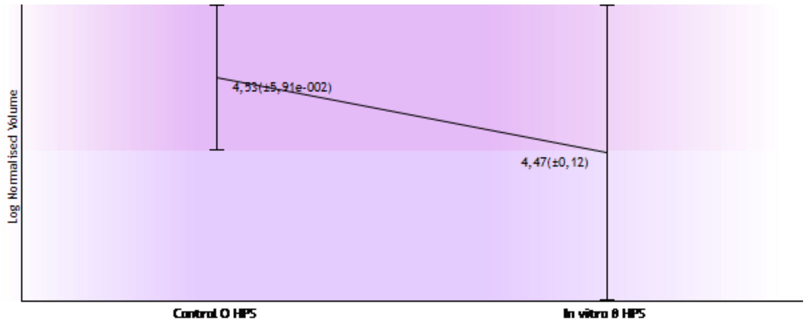

Identifier 81

Position (1167, 528)
Notes

Control O HPS	In vitro 8 HPS

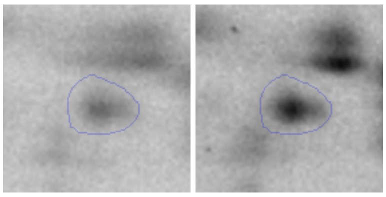

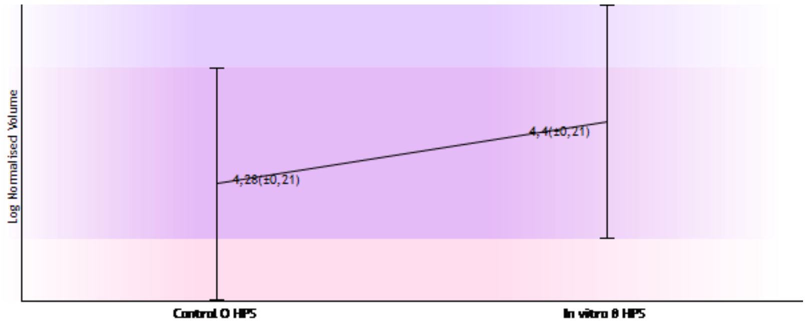

Identifier 83

Position (1239, 551)
Notes

Control O HPS	In vitro 8 HPS

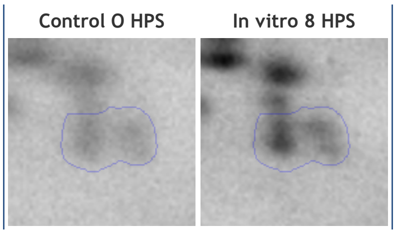

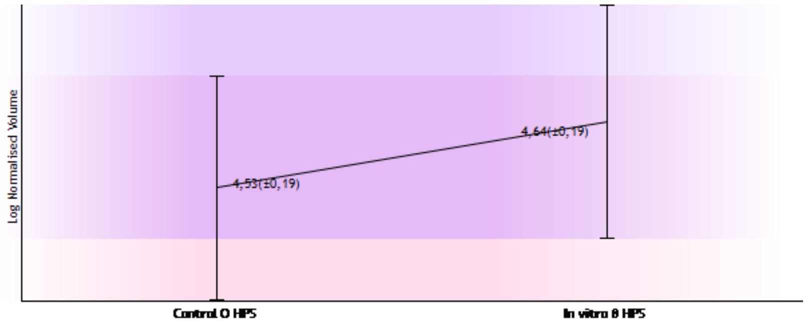

Identifier 84

Position (1139, 555)
Notes

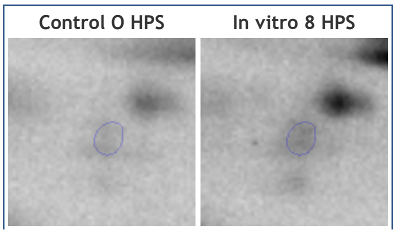

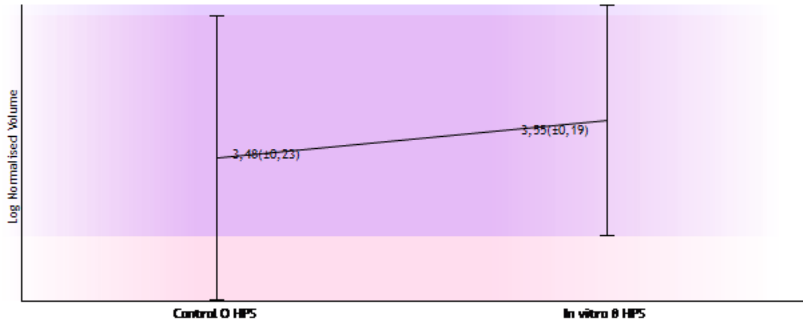

Identifier 86

Position (805, 574)
Notes

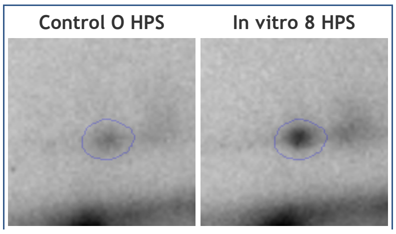

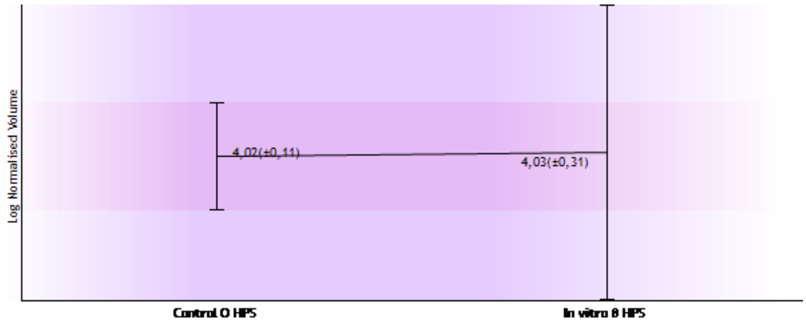

Identifier 88

Position (1383, 578)
Notes

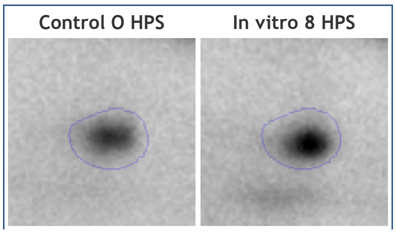

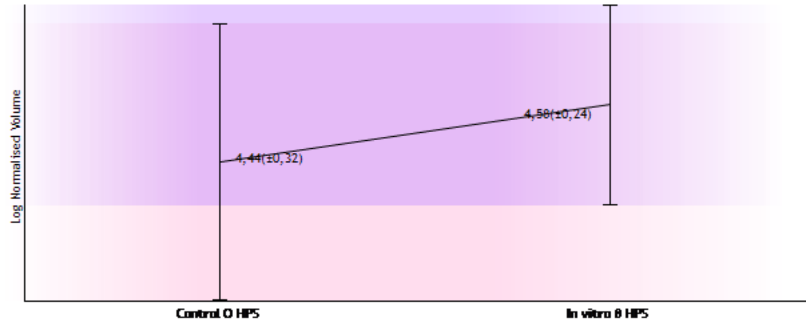

Identifier 90

Position (1539, 585)
Notes

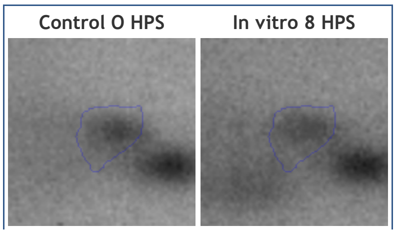

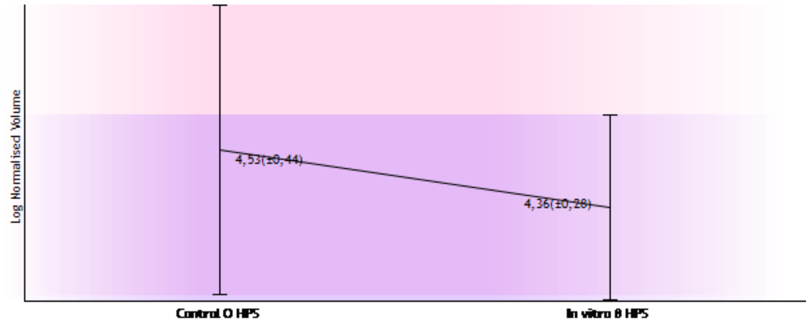

Identifier 91

Position (1031, 590)
Notes

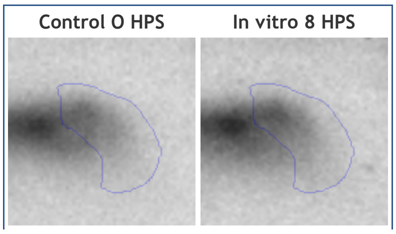

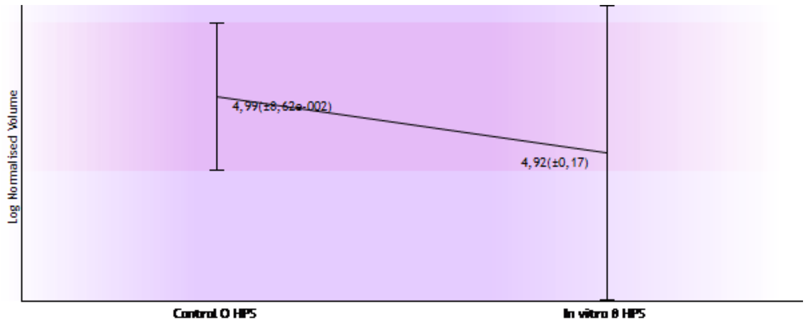

Identifier 92

Position (1702, 598)
Notes

Control O HPS	In vitro 8 HPS

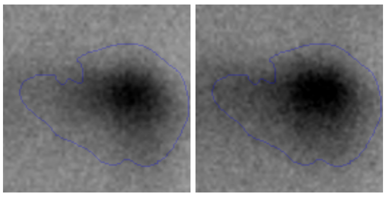

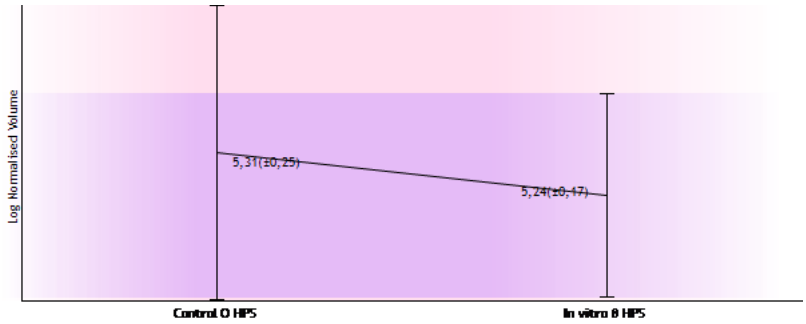

Identifier 93

Position (1569, 604)
Notes

Control O HPS	In vitro 8 HPS

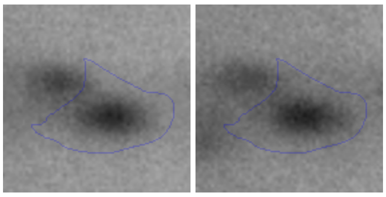

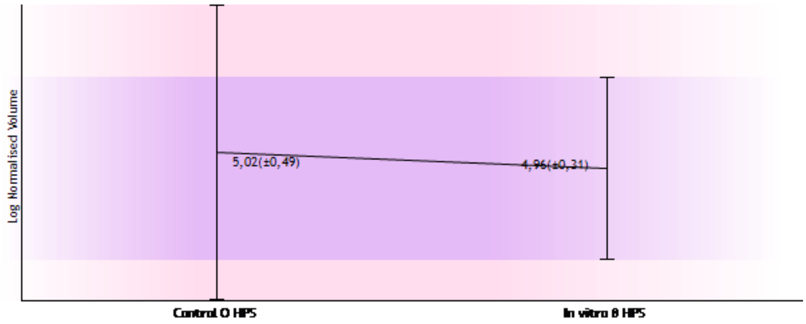

Identifier 94

Position (885, 617)
Notes

Control O HPS	In vitro 8 HPS

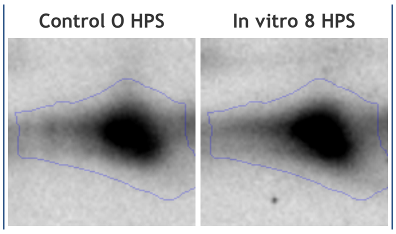

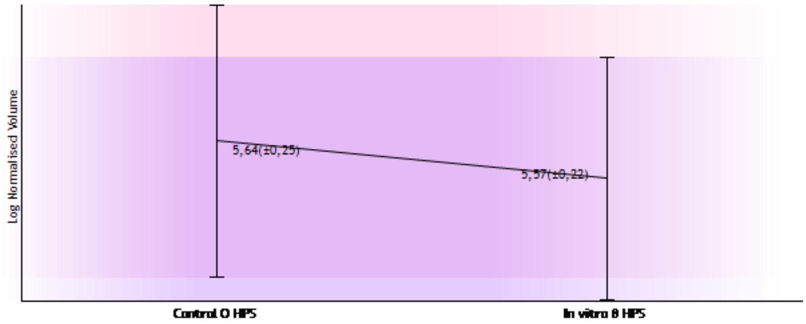

Identifier 95

Position (1028, 617)
Notes

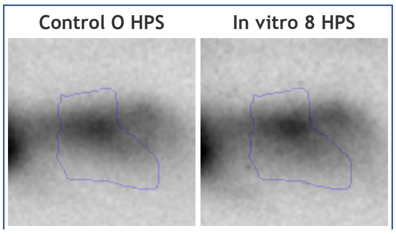

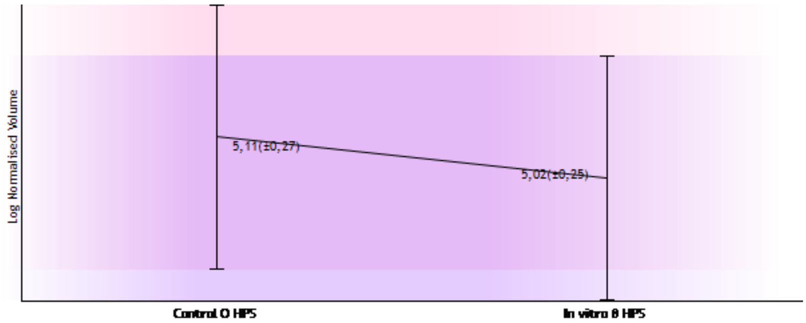

Identifier 100

Position (818, 632)
Notes

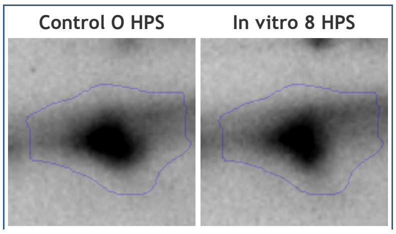

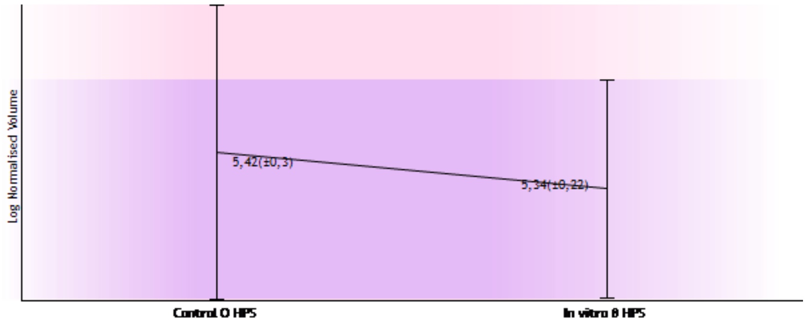

Identifier 102

Position (1367, 681)
Notes

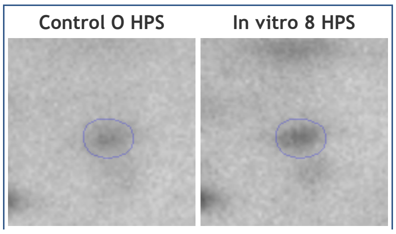

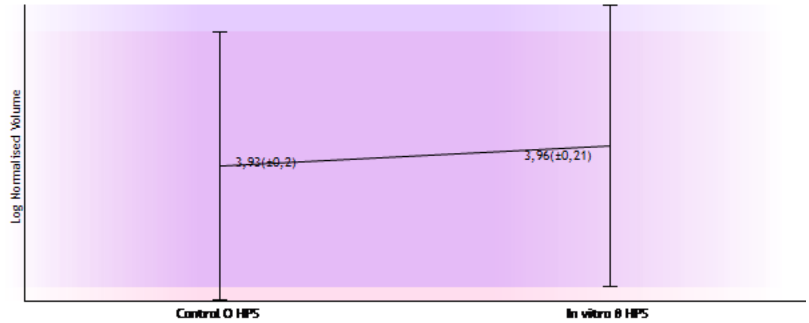

Identifier 104

Position (1256, 713)
Notes

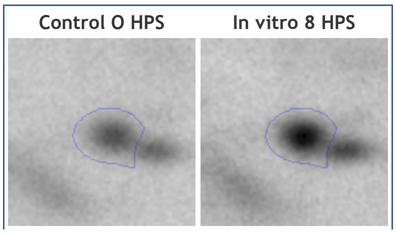

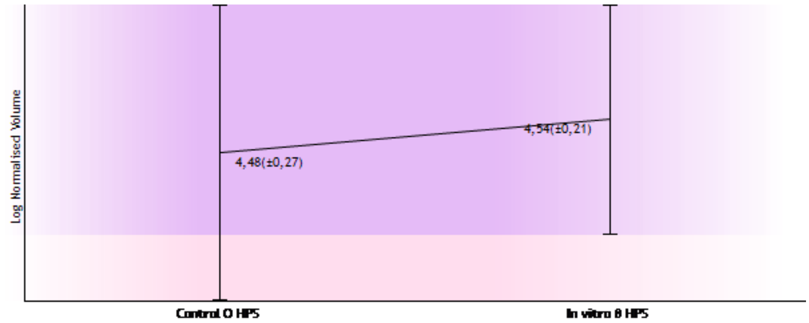

Identifier 106

Position (989, 722)
Notes

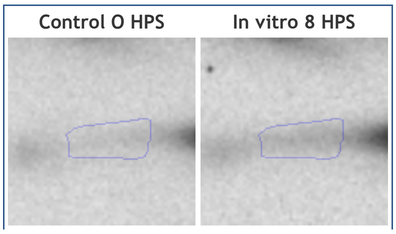

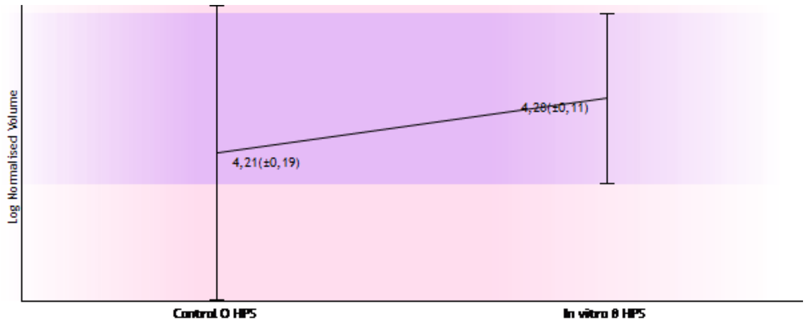

Identifier 108

Position (1291, 724)
Notes

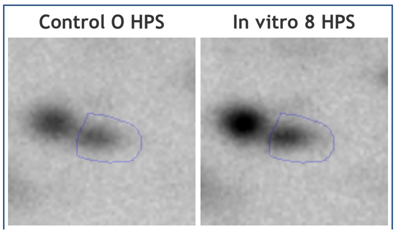

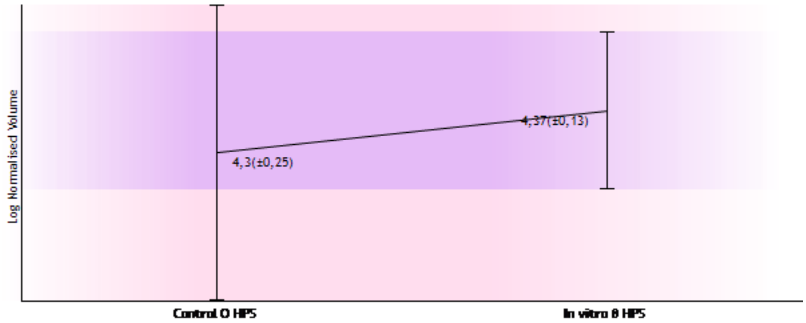

Identifier 109

Position (1037, 726)
Notes

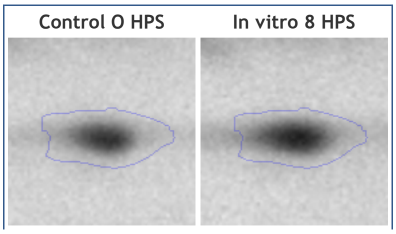

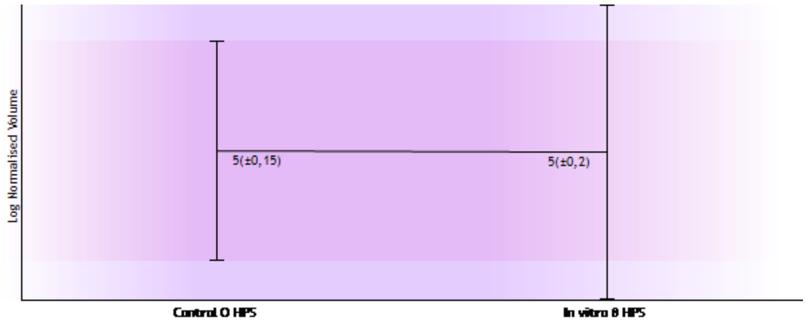

Identifier 110

Position (1143, 726)
Notes

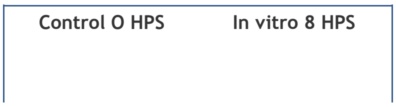

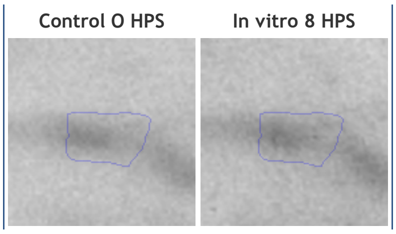

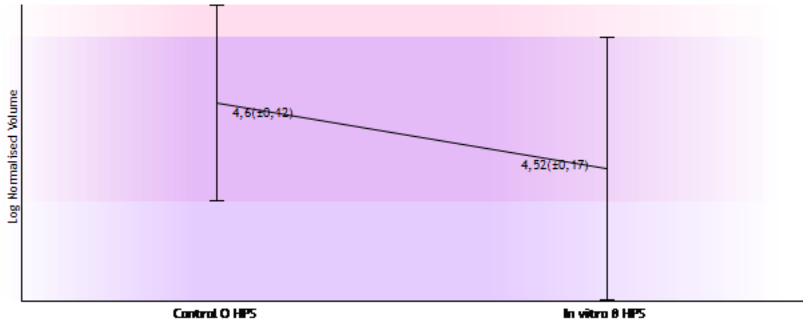

Identifier 111

Position (899, 732)
Notes

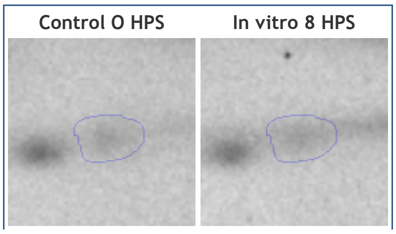

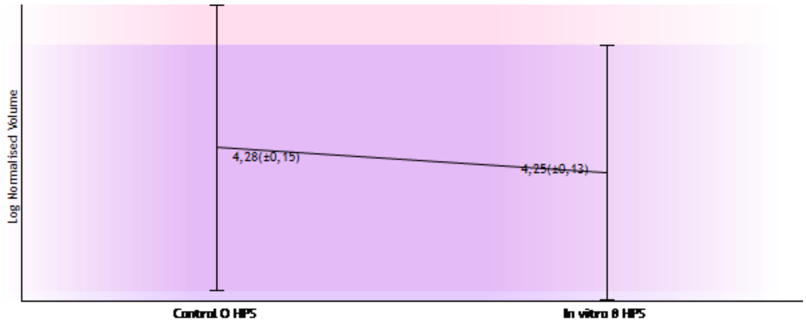

Identifier 112

Position (834, 737)
Notes

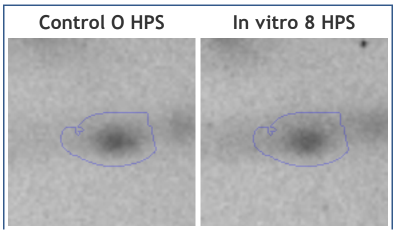

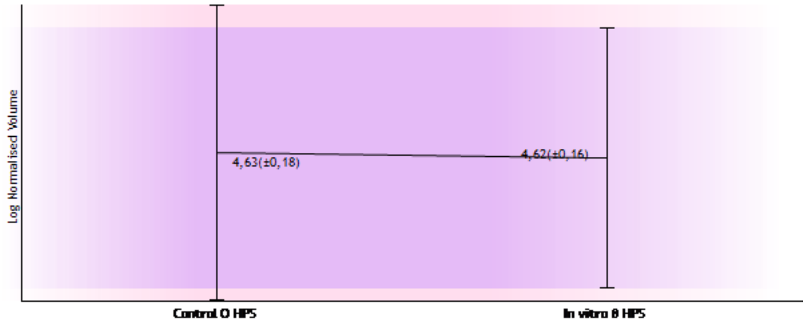

Identifier 113

Position (807, 742)
Notes

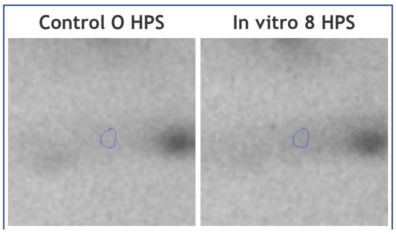

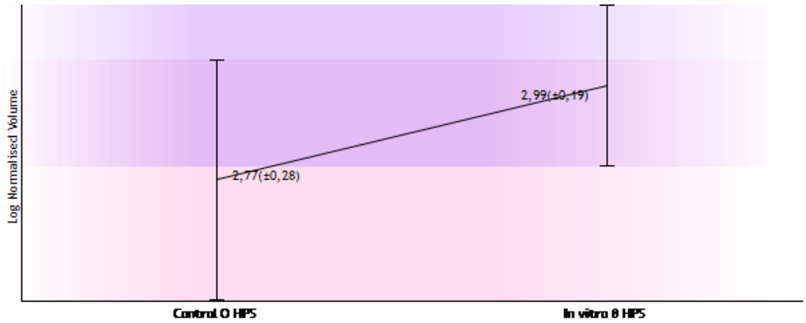

Identifier 116

Position (1214, 750)
Notes

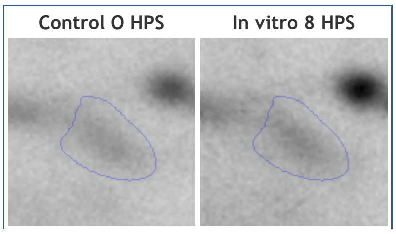

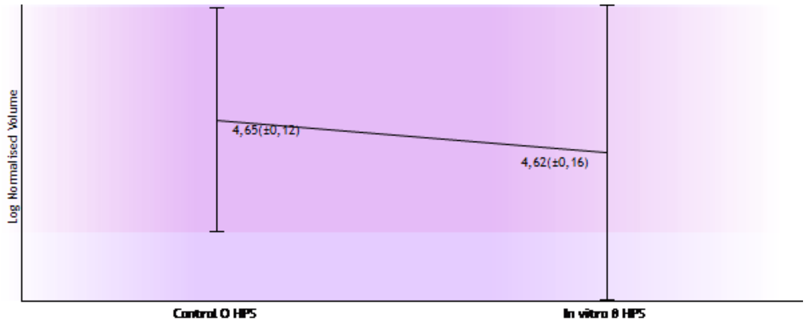

Identifier 118

Position (1470, 761)
Notes

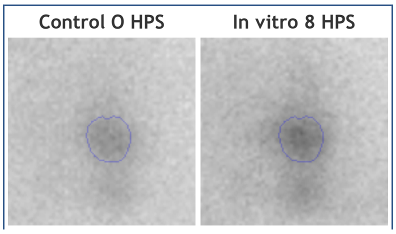

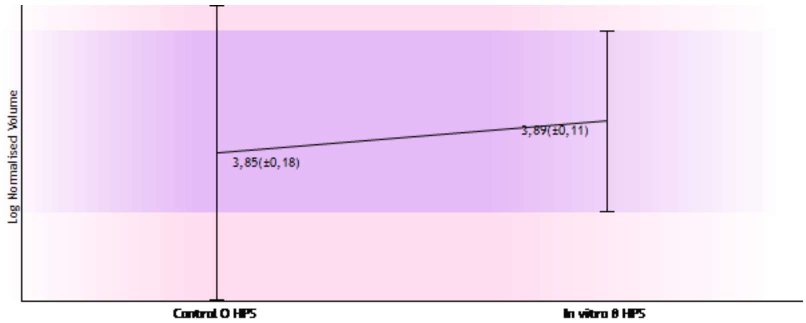

Identifier 122

Position (1586, 814)
Notes

Control O HPS	In vitro 8 HPS

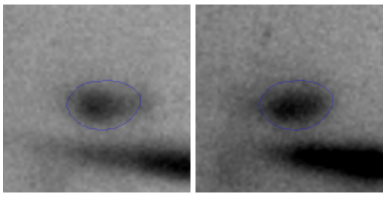

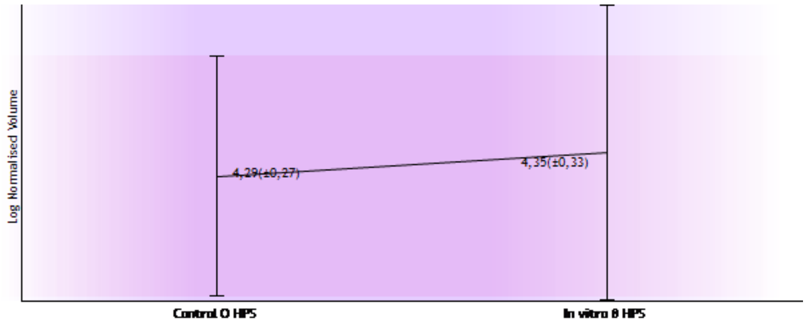

Identifier 123

Position (1707, 819)
Notes

Control O HPS	In vitro 8 HPS

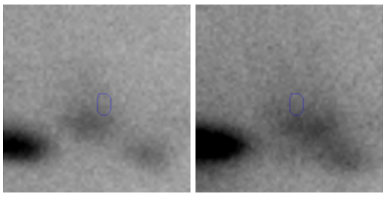

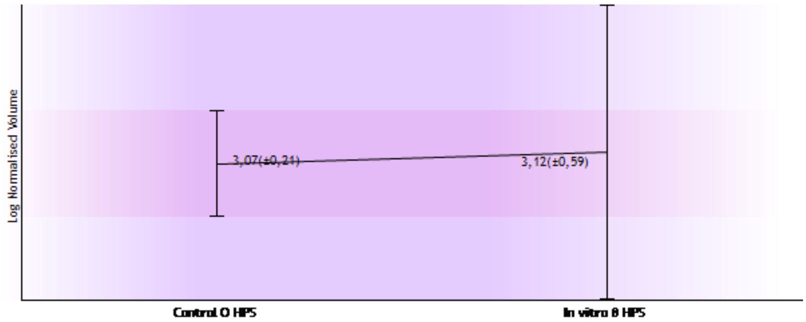

Identifier 125

Position (1710, 836)
Notes

Control O HPS	In vitro 8 HPS

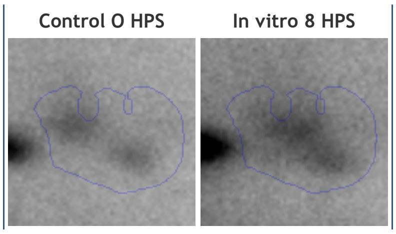

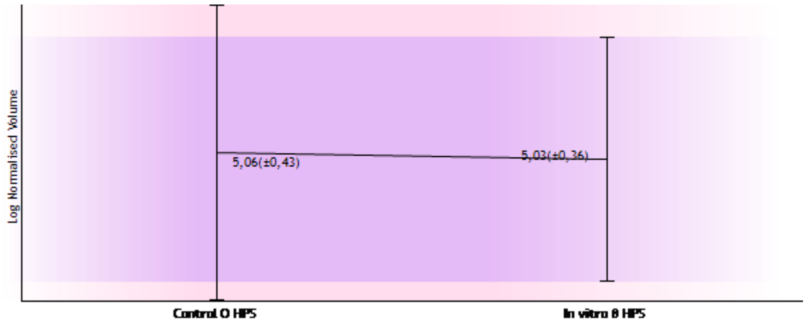

Identifier 132

Position (1754, 931)
Notes

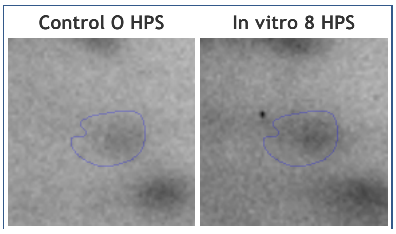

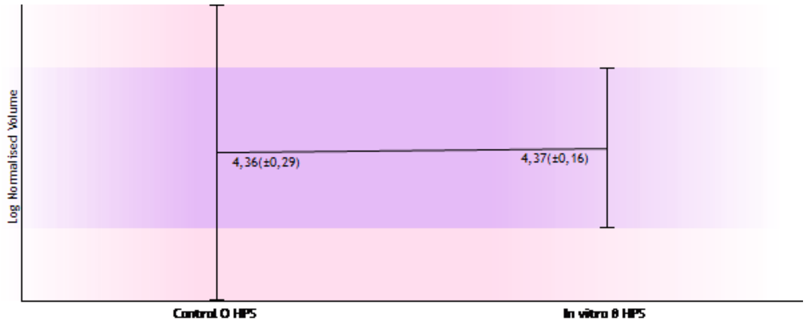

Identifier 137

Position (1788, 966)
Notes

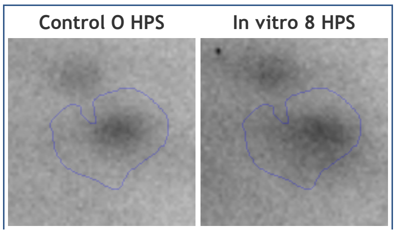

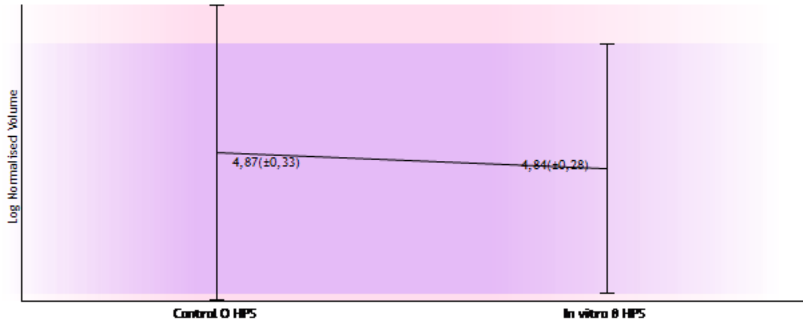

Identifier 143

Position (1812, 999)

Notes

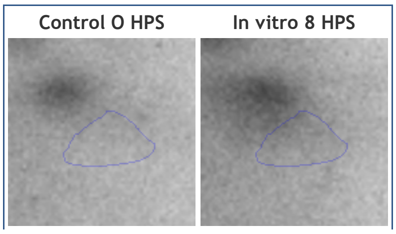

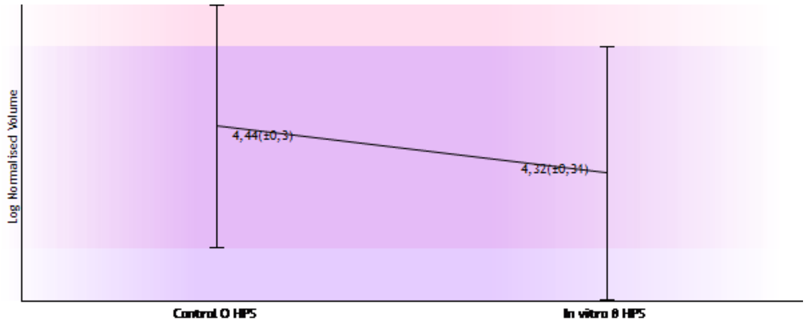

Identifier 149

Position (1850, 1144)

Notes

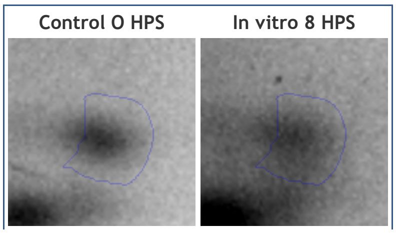

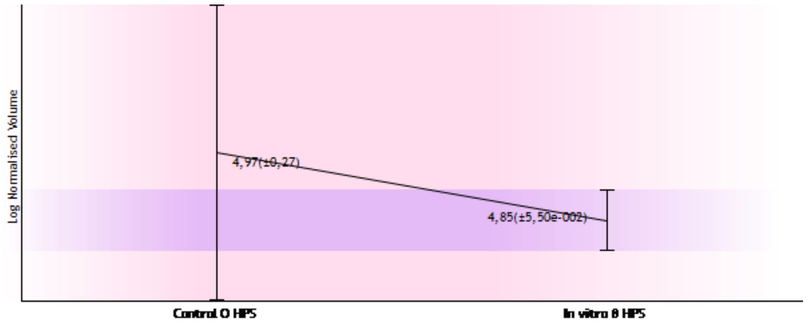

Identifier 156

Position (1788, 1193)

Notes

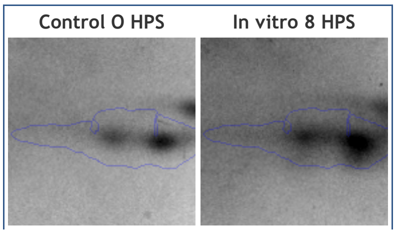

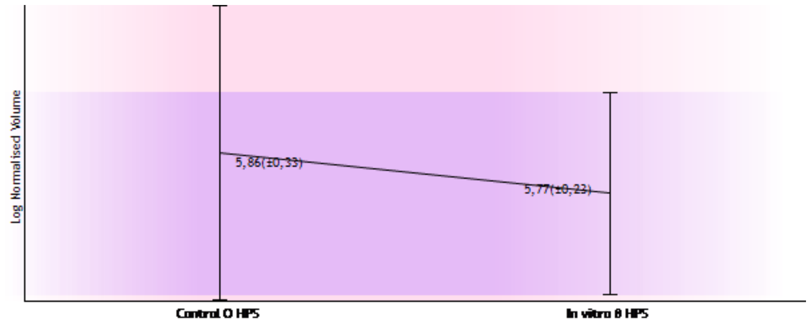
Log Normalised Volume
5,86(±0,33)
5,77(±0,23)
Control 0 HPS
In vitro 8 HPS
